# Novel compound heterozygous mutations of *MTHFR* Gene in a Chinese family with homocystinuria due to MTHFR deficiency

**DOI:** 10.1186/s12920-022-01408-4

**Published:** 2022-12-25

**Authors:** Yitong Lu, Shaozhi Zhao, Xiaohui He, Hua Yang, Xiaolei Wang, Chen Miao, Hongwei Liu, Xinwen Zhang

**Affiliations:** Xi’an People’s Hospital (Xi’an Fourth Hospital), Xi’an, China

**Keywords:** Homocystinuria, MTHFR deficiency, Cerebral dysplasia, Brain atrophy

## Abstract

**Background:**

Homocystinuria due to methylenetetrahydrofolate reductase (MTHFR) deficiency is a rare autosomal recessive disorder. The purpose of this study is to expand the mutation site of the *MTHFR* gene and provide genetic counseling for this family.

**Methods:**

A couple came to our hospital for pre-pregnancy genetic counseling. We collected the family history and detailed clinical information, then performed whole-exome sequencing, and analyzed the pathogenicity of the candidate mutations.

**Results:**

We found that the father of the proband had homocystinuria, the proband and his brother had low blood methionine levels at birth, and the brain MRI showed brain dysplasia. The third fetus was found to have a broadened triangle of the bilateral ventricle at 19 weeks of pregnancy. The compound heterozygous variants of c.602 A > C (p.His201Pro) and c.1316T > C (p.Leu439Pro) of the *MTHFR* gene in the first three fetuses were found by whole-exome sequencing. The heterozygous c.602 A > C variant of the *MTHFR* gene is a novel missense variant that has been submitted to the ClinVar with Variation ID 992,662.

**Conclusion:**

In consideration of the clinical phenotype, family history, and result of genetic testing, we speculated that both patients may have homocystinuria due to MTHFR deficiency. Homocystinuria due to MTHFR deficiency caused by compound heterozygous mutations composed of the *MTHFR* gene in this family may be associated with cerebral atrophy and cerebral dysplasia. The novel compound heterozygous mutations broaden the mutation spectrum of the *MTHFR* gene and enhance the application of genetic counseling and carrier screening in rare diseases.

## Introduction

Homocystinuria due to methylenetetrahydrofolate reductase (MTHFR) deficiency (OMIM: 236,250) is a rare autosomal recessive disorder [1, 2]. The Human *MTHFR* gene (OMIM: 607,093) is located on chromosome 1p36.22 and mainly consists of 12 exons(NM_005957.4) [2]. MTHFR can reduce 5,10-methylenetetrahydrofolate to 5-methylenetetrahydrofolate which acts as a methyl donor for the methylation of homocysteine (Hcy) to methionine (Met) [3]. The enzymatic reactions require flavin adenine dinucleotide (FAD) as a prosthetic group and nicotinamide adenine dinucleotide phosphate (NADPH) as an electron donor [4]. Both the folate and methionine cycles are essential products for the provision of cellular activity. Folate is the major cellular carrier in single carbon units, which is an essential material for the synthesis of purines and thymidine monophosphate. In the methionine cycle, the methylation of homocysteine generates methionine, which could further convert to S-adenosine methionine as a vital methyl donor for DNA, RNA, and proteins. The folate and methionine cycles intersect with the enzyme of MTHFR. Therefore, the deficiency of MTHFR restrains the methylation of Hcy to Met, resulting in an abnormally high level of total Hcy (tHcy) and a low level of Met in plasma. In this condition, homocystinuria could be caused by the disorder of folate metabolism and its FAD responsiveness [5, 6].

Up to now, there are 135 cases with *MTHFR* pathogenic gene variants in the Human Gene Mutation Database (HGMD), most of them are missense mutations [5]. Patients with homocystinuria due to MTHFR deficiency often suffer from diverse symptoms, like mental retardation, myelopathy, ataxia, and spasm. However, severe early-onset usually develops in infancy, and children with severe homocystinuria due to MTHFR deficiency often show diverse phenotypes, including eating difficulties, hypotonia, neurocognitive disorders, ventricular dilatation, hydrocephalus, brain atrophy, microcephaly, and epilepsy [7].

In this study, we reported a family case with homocystinuria. To determine the likely pathogenic gene variants in the family pedigree, we performed whole-exome sequencing in this study. We speculate that the compound heterozygous variants of c.602 A > C (p.His201Pro) and c.1316T > C (p.Leu439Pro) of the *MTHFR* gene may be new genetic variants associated with homocystinuria due to MTHFR deficiency.

## Patients and methods

### Patients

This study was conducted in strict accordance with the principles of the Helsinki Declaration. The patients and their families signed consent for using their data, and the work was approved by the ethical review committee of our hospital.

The proband (II-1) was a 2-month-old female infant, who was admitted to the hospital with impaired consciousness, paroxysmal shortness of breath, and vomiting (occurred 2 times within 12 h of observation). Neonatal screening results showed 6.98 µmol/L of Met (reference value 7.5–45 µmol/L), 0.22 µmol/L of Met/Cys (reference value 0.5-4 µmol/L), and 0.03 µmol/L of Met/Leu (reference value 0.06–0.24 µmol/L). The chest radiograph (CR) showed bronchitis. Computed tomography of her head revealed an enlarged bilateral frontotemporal sub-arachnoid space, slightly enlarged lateral ventricles, left lateral ventricle subependymal cyst, and bilateral maxillary sinusitis. Upper gastrointestinal angiography indicated gastric volvulus. The symptoms did not improve after 1 month of treatment, as evidenced by the examination of the head magnetic resonance imaging (MRI) revealing traffic hydrocephalus, bilateral frontotemporal apical space enlargement, and a left lateral ventricle subependymal cyst. After the following treatment for another month, the examination of the head MRI revealed a left ventricle subependymal hemorrhage and cerebral atrophy. The patient eventually died as a consequence of multiple symptoms, possibly including bronchopneumonia-induced type I respiratory failure and atelectasis, communicating hydrocephalus, and cerebral atrophy.

**Fig. 1:**
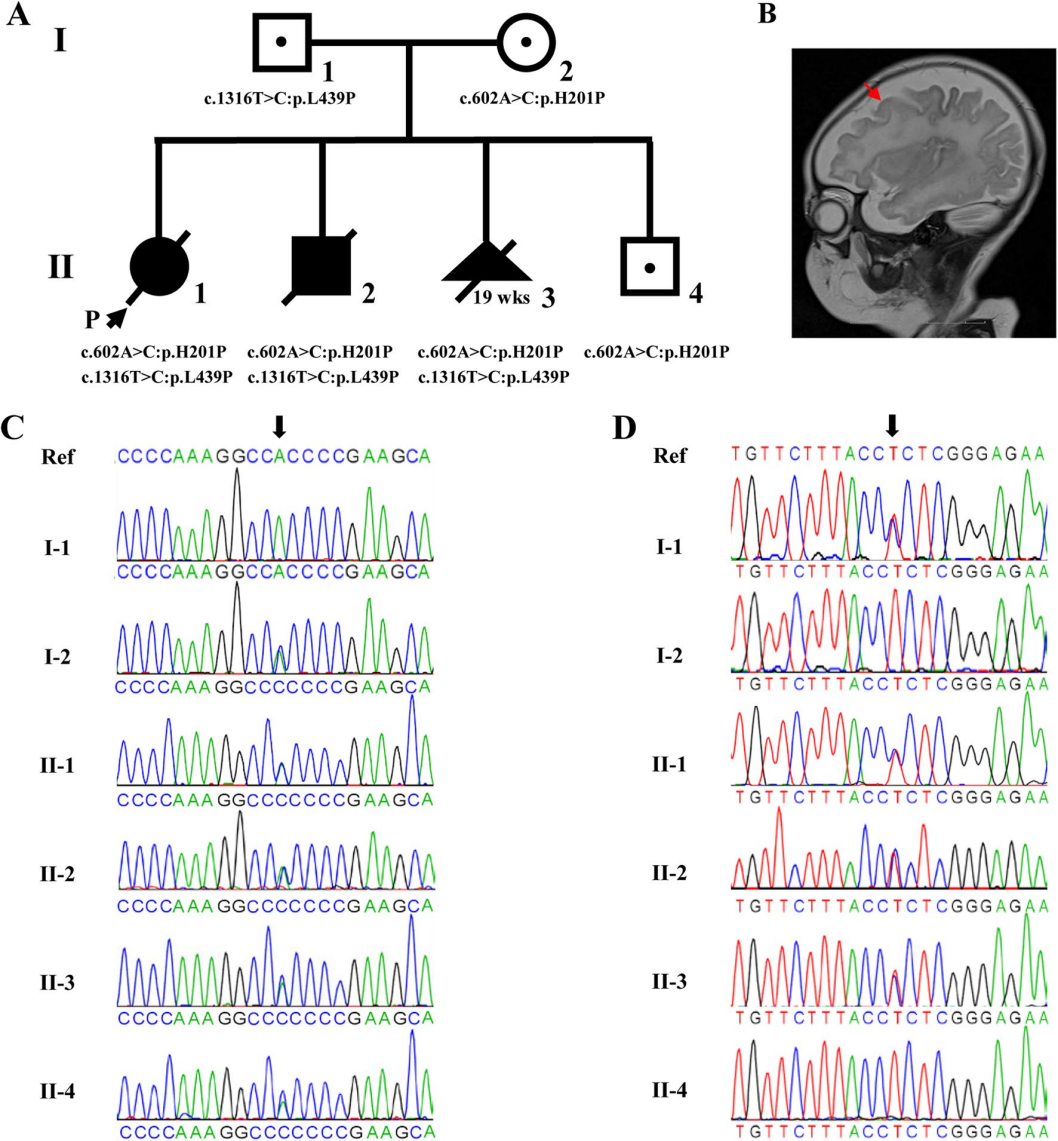
Clinical features and genetic sequencing data of the patients. **A** The pedigree of the family. **B** Head MRI of II-2. **C** *MTHFR* NM_005957.4 c.602 A > C (p.H201P). **D** *MTHFR* NM_005957.4 c.1316T > C (p.L439P)

The second child (II-2) was a 2-month-old male infant who was admitted to the hospital after choking on milk for 1 week and showed shortness of breath during the day. CR revealed a patchy-like high-density shadow in both lungs. Head MRI T2WI showed increased white matter signal, widening and deepening of the cerebral cortex, thinning of the cortex, and widening of subdural space, indicating cerebral dysplasia (Fig. 1B**)**. Inherited metabolic diseases determination report showed 7.93 µmol/L of Met (reference value 8–35 µmol/L), 0.18 µmol/L of Met/Phe (reference value 0.2–0.6), and 242.58 µmol/L of Glu (reference value 45–200 µmol/L). Chromosomal analysis revealed a normal karyotype (46, XY). After one month, the patient died of multiple symptoms, including severe bronchopneumonia, cerebral dysplasia, bronchopulmonary dysplasia, and moderate anemia.

The third child (II-3) was a fetus at 19 weeks of gestation, of whom MRI examination showed bilateral lateral ventricle trigonometry enlargement. The parents of the fetus decided to choose a medical abortion to avoid birth defects.

The fourth male child (II-4) was delivered by cesarean section at 40 weeks of gestation. The newborn weighed 4060 g and was diagnosed with macrosomia. There was no perinatal abnormality (such as hypoxia and asphyxia) at birth. The level of homocysteine in the postnatal peripheral blood was 10.6 µmol/L (reference value 0–15 µmol/L). Four months later, the homocysteine level was found to be elevated to 16.4 µmol/L (reference value 0–15µmol/L) but the levels of complete blood count, vitamin B12, folic acid, and ferritin were all normal. The father of the proband had a slight increase in peripheral blood homocysteine (Hcy: 27.6 µmol/L) and no other obvious clinical manifestations. It was improved after folic acid and vitamin B12 supplementation but returned to a higher Hcy level (Hcy: 52.2 µmol/L) after withdrawal. The proband’s mother is in good health condition. The pedigree chart of the family was presented in Fig. 1 A.

### Library preparation

Firstly, genomic DNA was extracted from 200µL peripheral blood, using a Qiagen DNA Blood Midi/Mini kit (Qiagen GmbH, Hilden, Germany). About 50 ng of genomic DNA was interrupted to around 200 bp fragments by the enzyme. The DNA fragments were end-repaired by adding one A base at the 3’end.

Secondly, the DNA fragments were ligated with barcoded sequencing adaptors, and ligated fragments (about 320 bp) were collected by XP beads. After PCR amplification, the DNA fragments were hybridized and captured by NanoWES(Berry Genomics, China) according to the manufacturer’s Protocol. The hybrid products were eluted and collected, and then subjected to PCR amplification and purification. Next, the libraries were quantified by qPCR, and size distribution was determined using an Agilent Bioanalyzer 2100 (Agilent Technologies, Santa Clara, CA, USA). Finally, the Novaseq 6000 platform (Illumina, San Diego, USA) with 150 bp pair-end sequencing mode was used for sequencing the genomic DNA samples of the family. Raw image files were processed using CASAVA v1.82 for base calling and generating raw data [8].

### Data analysis

The sample genomes were aligned to the human reference genome (hg19/GRCh37) using the Burrows-Wheeler Aligner tool and PCR duplicates were removed by using Picard v1.57 (http://picard.sourceforge.net/). Verita Trekker^®^ Variants Detection System by Berry Genomics and the third-party software GATK (https://software.broadinstitute.org/gatk/) were employed for variant calling. Variant annotation and interpretation were performed with ANNOVAR (Wang, et al., 2010) and the Enliven® Variants Annotation Interpretation System authorized by Berry Genomics.

Annotation databases mainly included:

(i) Human population databases, including gnomAD (http://gnomad.broadinstitute.org/), the 1000 Genome Project (http://browser.1000genomes.org), Berrybig data population database, dbSNP (http://www.ncbi.nlm.nih.gov/snp) etc.;
(ii) Databases of in silico prediction algorithms, including SIFT (http://sift.jcvi.org), FAT-HMM (http://fathmm.biocompute.org.uk), MutationAssessor (http://mutationassessor.o-rg), CADD (http://cadd.gs.washington.edu), SPIDEX (Xiong et al., Science 2015);
(iii) Disease and phenotype databases, including OMIM (http://www.omim.org), ClinVar (http://www.ncbi.nlm.nih.gov/clinvar), HGMD (http://www.hgmd.orgO (http://hpo.jax.org/app/). The variants were classified into five categories: pathogenic, likely pathogenic, uncertain significance, likely benign, and benign, according to the American College of Medical Genetics and Genomics (ACMG) guidelines for the interpretation of genetic variants. Variants with minor allele frequencies (MAF) < 1% in the exonic region or with splicing impact were taken for deep interpretation based on the ACMG category, evidence of pathogenicity, clinical synopsis, and inheritance model of the associated disease. Sanger sequencing was further used to identify the presence of the pathogenic variants in the family members.

## Results

The couple came to our hospital when they were pregnant with their third child. To determine the likely pathogenic gene variants in the family pedigree, we performed whole-exome sequencing in patient II-3 and reanalyzed the whole-exome data of II-1, and II-2. The genetic status of the parents was detected by Sanger sequencing. It revealed compound heterozygous c.602 A > C (p.His201Pro) variant in exon 5 and c.1316T > C (p.Leu439Pro) variant in exon 8 of the *MTHFR* gene in the patients of II-1, II-2, and II-3. The Sanger sequencing also indicated that the c.602 A > C variant was inherited from the mother and the c.1316T > C variant was inherited from the father (Fig. 1 C, D**)**. One year later, we performed whole-exome sequencing in patient II-4, and we found he was only a carrier of c.602 A > C. Both identified heterozygous variants were missense mutations, in which the genetic variant of c.602 A > C is a newly reported mutation while the variant of c.1316T > C was previously reported in adults but not in infants with severe homocystinuria due to MTHFR deficiency[9]. The newly discovered variant sites have been submitted to the ClinVar website, Variation ID: 992,662, https://www.ncbi.nlm.nih.gov/clinvar/variation/992662.

To further evaluate the pathogenicity of the compound heterozygous mutation, several amino acid missense mutation prediction programs, e.g. SIFT, PROVEAN, PolyPhen-2, Mutation Taster, Revel, and ClinPred were used to predict the deleteriousness of variants. Differently, Polyphen-2 showed the genetic variant of c.602 A > C and c.1316T > C were likely damaging, while SIFT, PROVEAN, and Mutation Taster showed that both heterozygous variants of c.602 A > C and c.1316T > C were damaging. The Revel score and ClinPred score of the variant of c.602 A > C were 0.887 and 0.999, the Revel score and ClinPred score of the variant of c.1316T > C were 0.835 and 0.995, they all show that these two missense mutations are damaging.

According to the ACMG guidelines, the variant c.1316T > C (p.Leu439Pro) should be classified as likely pathogenic by fulfilling the standard PS3 + PM2 + PP3. The rs545086633 SNP (p.Leu439Pro) results in an L439P substitution in MTHFR protein and drastically decreases mutant protein expression by promoting proteasomal degradation (PS3) [9]. The mutation was not found in the human exome database (ExAC) and the population genome mutation frequency database (gnomAD). The frequency in the reference population 1000 genomes (1000G) was 0.001 (PM2). In silico prediction analyses predicted a deleterious effect on gene product for this variant (PP3).

According to the ACMG guidelines, the variant c.602 A > C (p.His201Pro) should be classified as likely pathogenic by fulfilling the standard PM2 + PM3 + PP1 + PP3. The variant was not found in ExAC, 1000G, and gnomAD (PM2). This variant forms compound heterozygosity (in trans state) with likely pathogenic c.1316T > C variation site (PM3). In the family, two affected family members (II-1 and II-2) with compound heterozygous variants were co-segregation with disease. Bilateral lateral ventricle trigonometry enlargement was also observed in II-3 with compound heterozygous variants. We were unable to observe further phenotypes due to the fetus was induced. While II-4 only carry one variant was healthy (PP1)[10]. Multiple software predicted its conservatism, the results showed that the site was evolutionarily conserved and had potential functional effects; the protein function was predicted by SIFT and LRT, and the results showed that it was harmful (PP3).

**Fig. 2:**
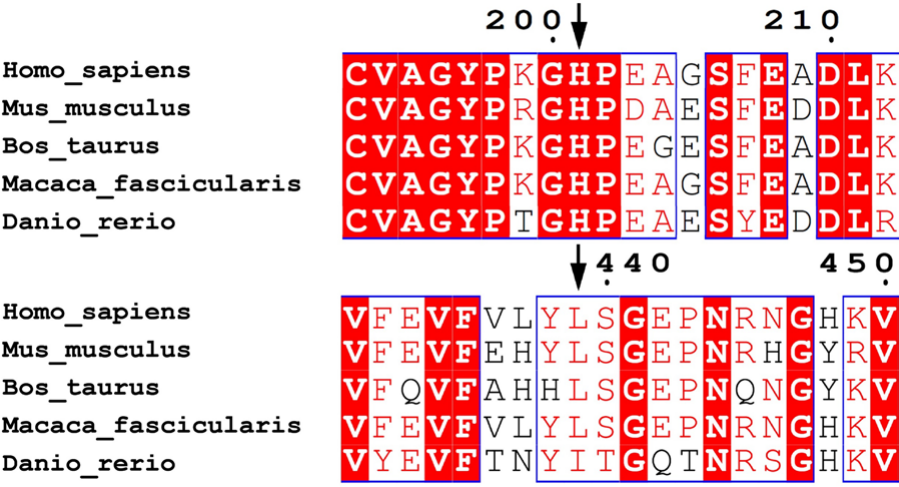
Clustal W alignment of MTHFR proteins among the representative species at the sites of p.His201 and p.Leu439

**Fig. 3:**
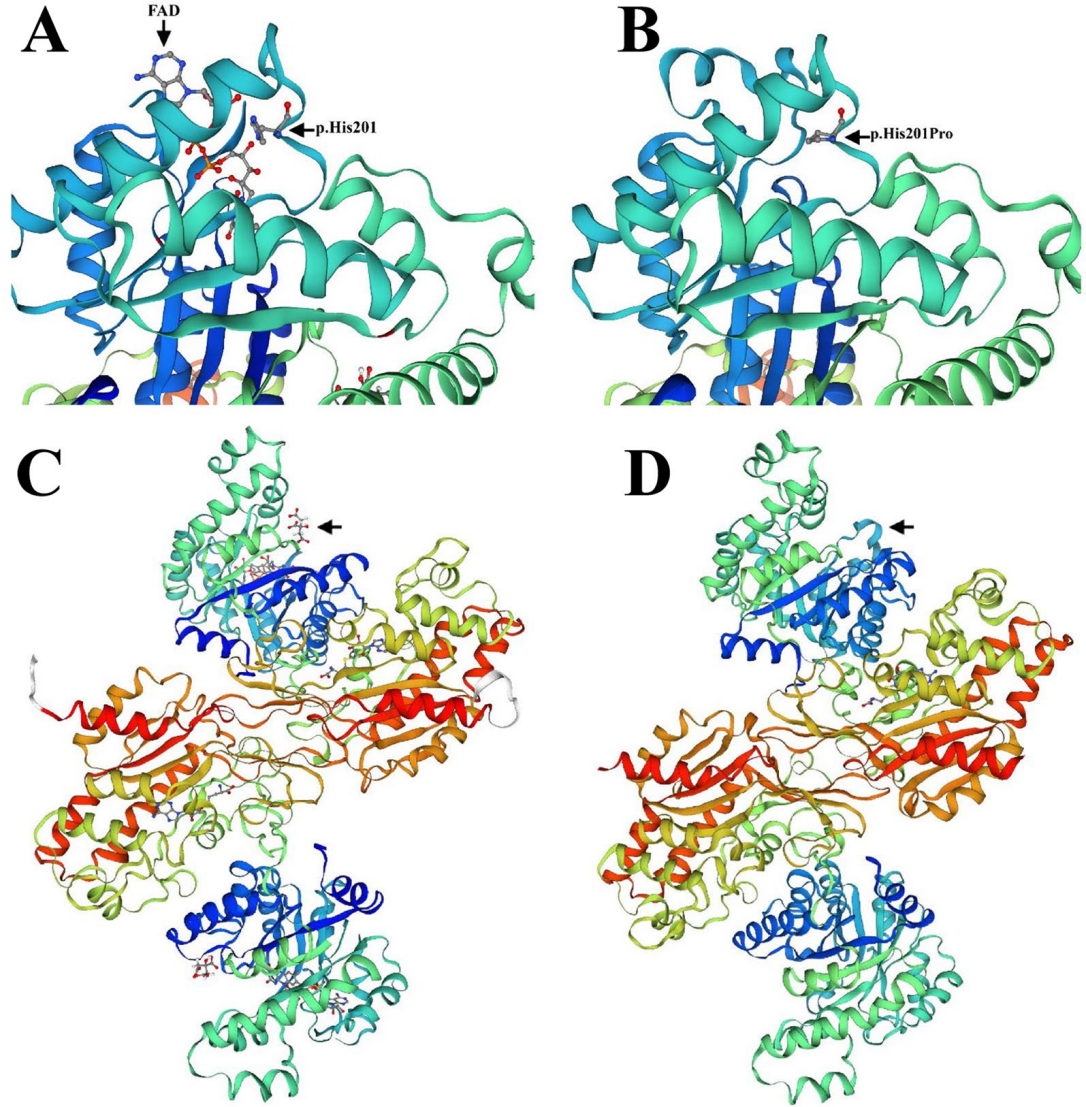
Modeling of the Human MTHFR protein. **A** p.His201 and FAD. **B** p.His201Pro. **C** Modeling of the wild MTHFR protein. **D** Modeling of the MTHFR protein variant

The MUSCLE (https://www.ebi.ac.uk/Tools/msa/muscle/) was used to compare the amino acid sequence of the *MTHFR* gene for multiple species and found two missense variants are highly conserved across different species (Fig. 2**)**. According to the identified *MTHFR* gene variants, the SWISS-MODEL (https://swissmodel.expasy.org/interactive) was used to generate the three-dimensional (3D) models of the protein variants for functional structure prediction compared with the known Human MTHFR protein in the current database (https://swissmodel.expasy.org/repository/uniprot/P42898) (Fig. 3 A, 3B, 3 C, 3D**)**. The comparison suggested that the MTHFR protein variant may lose its FAD-binding domain, resulting in the loss of its binding ability with FAD-during the methylation of Hcy.

## Discussion

There is a wide range of clinical manifestations in homocystinuria due to MTHFR deficiency. However, severe early-onset usually develops in infancy, and children with severe homocystinuria due to MTHFR deficiency often show diverse phenotypes, including eating difficulties, hypotonia, neurocognitive disorders, ventricular dilatation, hydrocephalus, brain atrophy, microcephaly, and epilepsy [1]. In this study, severe homocystinuria due to MTHFR deficiency occurred 2 months after birth for two children, accompanied by initial symptoms, like impaired consciousness, vomiting milk, and shortness of breath. In the development of the disease, the functions of the nervous system and respiratory system of the two patients significantly deteriorated. MRI in the two patients showed hydrocephalus, cerebral dysplasia, brain atrophy, and intracranial hemorrhage, as typical manifestations of severe homocystinuria due to MTHFR deficiency [11–13]. Out of 30 patients with severe homocystinuria due to MTHFR deficiency, Huemer et al. found 15 with brain atrophy, 13 with Leukodystrophy/dysplasia of the brain myelin sheath, and 9 with ventricular enlargement/hydrocephalus in their MRI examination [1]. Surtees et al. demonstrated that demyelination caused by defective methylation of homocystinuria due to MTHFR deficiency could be associated with the deficiency of S-adenosine methionine in cerebrospinal fluid, with the evidence of the remyelination achieved by the recovery of S-adenosine methionine [14]. In our study, the proband (II-1) and II-2 were found to have a slight decrease in the blood methionine level during neonatal screening and did not receive any treatment. Consequently, this fact makes doctors hard to determine the onset age of homocystinuria due to MTHFR deficiency for patients (a chance of misdiagnosis) and dismiss the link with possible gene variations in *MTHFR*. Although a lot of examinations and clinical treatments have been conducted for children, unfortunately, their homocysteine and methionine have not been tested, and the opportunity for diagnosis and symptomatic treatment may be missed. The investigation of a genetic defect in homocystinuria due to MTHFR deficiency could be important for optimizing clinical treatment strategies. For instance, the finding on the *MTHFR* defects in homocystinuria due to MTHFR deficiency helps to explain why the supplementation of B12 did not improve the deteriorating nervous system for homocystinuria due to MTHFR deficiency patients and the use of CH3-THF cannot make the neurotransmitter normalized, whereas the use of betaine can effectively prevent the death of patients with early-onset of homocystinuria due to MTHFR deficiency and improve the dysplasia symptoms of the nervous system by remethylating of Hcy to Met [15, 16].

Human MTHFR is a spherical structural protein consisting of a homodimer with identical subunits, each of which carries an N-terminal catalytic domain (aa 1-356), a C-terminal regulatory domain (aa 363–656), and a short-chain connection domain in the middle (aa 357–362) [3, 17]. The N-terminal regulatory domain consists of an 8α/8β Tim Barrel structure and three additional α helices as a conserved catalytic domain that binds 5-methyltetrahydrofolate NADPH and FAD to complete the catalytic reaction. The C-terminal catalytic domain consists of two five-stranded β-sheet structures arranged in the core region, as a regulatory domain that binds to its allosteric inhibitor (S-adenosine methionine) to regulate enzyme activity for sustaining the level of methionine in the cell. Another study reported that severe *MTHFR* deficiency could be associated with genetic variants of the FAD binding site (e.g. Thr129, Arg157, Ala175, and Ala195) in the human MTHFR protein [18]. In this study, we identified a novel gene variant of c.602 A > C (p.His201Pro) in the family with homocystinuria due to MTHFR deficiency, which results in a missense mutation on exon 5 of the *MTHFR* gene, and the 201 His is one of the FAD-binding sites of the MTHFR protein. In the prediction analysis of MTHFR protein variants, we found that the alteration of p.His201pro may disable the binding ability of MTHFR to the FAD prosthetic group, thus reducing the enzyme activity. Meanwhile, we also detected another heterozygous variant c.1316T > C (p.Leu439Pro) in *MTHFR* in this family case. Liu et al. previously demonstrated that severe homocystinuria due to MTHFR deficiency patients with tardive dyskinesia carried two MTHFR deficiency-associated SNPs (rs748289202 p.Arg335His and rs545086633 p.Leu439Pro) on the *MTHFR* [9], and their further investigation confirmed that rs545086633 was responsible for the missense mutation of p.Leu439Pro in the C-terminal regulatory domain of MTHFR protein, and the proteasome degradation caused by the mutation led to suppress the expression of MTHFR protein.

Another noteworthy problem is that the proband’s father only carries a heterozygous variant of c.1316T > C in the *MTHFR* gene but the content of homocysteine in peripheral blood increases slightly. This situation has not been found in previous reports [9]. It was improved after folic acid and vitamin B12 supplementation but returned to a higher Hcy level after withdrawal. Consistent with previous reports, folic acid and vitamin B12 are not effective in patients with MTHFR deficiency [16].

The couple came to our hospital when they were pregnant with II-3 and received a comprehensive prenatal diagnosis. After reanalyzing the whole-exome data of II-1 and II-2, we found that the functional test of c.1316T > C (p.Leu439Pro) had been published [9]. The pathogenicity of c.1316T > C (p.Leu439Pro) was changed from a variant of uncertain significance to likely pathogenic, which determined the pathogenicity of the compound heterozygous mutation. Finally, the patient chose to have an abortion (II-3) and successfully obtained a healthy baby (II-4). Therefore, data reanalysis and comprehensive prenatal diagnosis are very important for screening and prenatal diagnosis of rare diseases. In consideration of the clinical phenotype, family history, and result of genetic testing, we speculated that both patients had MTHFR deficiency due to the compound heterozygous variants of c.602 A > C (p.His201Pro) and c.1316T > C (p.Leu439Pro) on the *MTHFR* gene.

## Conclusion

In conclusion, this study performed extensive gene sequencing analysis with existing biochemical screening and imaging examination in a Chinese family with multiple severe homocystinuria due to MTHFR deficiency cases and found novel compound heterozygous gene variants, c.602 A > C (p.His201Pro) and c.1316T > C (p.Leu439Pro) of the *MTHFR* gene. The new pathogenic gene variants enrich the mutation spectrum of the *MTHFR* gene and contribute to improving the diagnosis of homocystinuria due to MTHFR deficiency, considering that homocystinuria due to MTHFR deficiency remains difficult to be determined depending on clinical phenotypes. The outcomes will also benefit the genetic counseling of homocystinuria due to MTHFR deficiency in the future.

## Data Availability

The details of the variant analyzed during the current study are available in the ClinVar repository, under the Accession Number: VCV000992662.1.(https://www.ncbi.nlm.nih.gov/clinvar/variation/992662/?new_evidence=true) The raw datasets generated during the current study are not available for the time being because personal privacy may be compromised. If necessary, permission to access the original sequencing data can be obtained through the corresponding author.

## Abbreviations

ACMG: American College of Medical Genetics and Genomics
CR: Chest radiograph
FAD: Flavin adenine dinucleotide
Hcy: Homocysteine
HGMD: Human Gene Mutation Database
MAF: Minor allele frequencies
MRI: Magnetic resonance imaging (MRI)
Met: Methionine
MTHFR: Methylenetetrahydrofolate reductase
NADPH: Nicotinamide adenine dinucleotide phosphate.
